# Preparation and formation mechanism study of the long-term stable foamed sodium carboxymethyl cellulose loaded material

**DOI:** 10.1098/rsos.241354

**Published:** 2025-03-12

**Authors:** Mingmin Zhang, Xianzhe Xu, Guangyi Jiang, Zhecong Ying, Haiqiao Zhu, Chen Zuo

**Affiliations:** ^1^Institute of Radiochemistry, China Institute of Atomic Energy, Beijing 102413, People’s Republic of China

**Keywords:** foamed CMC, macroscopic mechanism, microscopic mechanism, loaded material, reducible C1

## Abstract

A foamed sodium carboxymethyl cellulose (CMC) material was prepared under nitric acid conditions. Unlike traditional CMC materials, this foaming method is straightforward and does not require additional foaming agents. Owing to its high stability and load capacity, the foam can realize long-term quantitative storage and load a variety of metal ions; therefore, it has broad application prospects in the field of loaded materials for metal ions. In this work, infrared spectroscopy and nuclear magnetic resonance (NMR) spectroscopy were used to explore the interaction between CMC and nitric acid in the foam under these conditions. The mechanism of foam formation was reasonably explained. Infrared spectra reveal the hydrolysis of the cellulose framework by nitric acid. Based on experimental observations during preparation and NMR analysis, it is explained that nitric acid activates glucose units’ C1 (no. 1 carbon in glucose unit) in CMC, leading to the formation of reducible terminal groups. Additionally, as the concentration of nitric acid increases during solution evaporation, a fraction of these reducible terminal groups undergo oxidation by nitric acid, resulting in gas production and subsequent expansion of the system, ultimately forming a foamed structure upon complete drying.

## 1. Introduction

**Figure 1.**
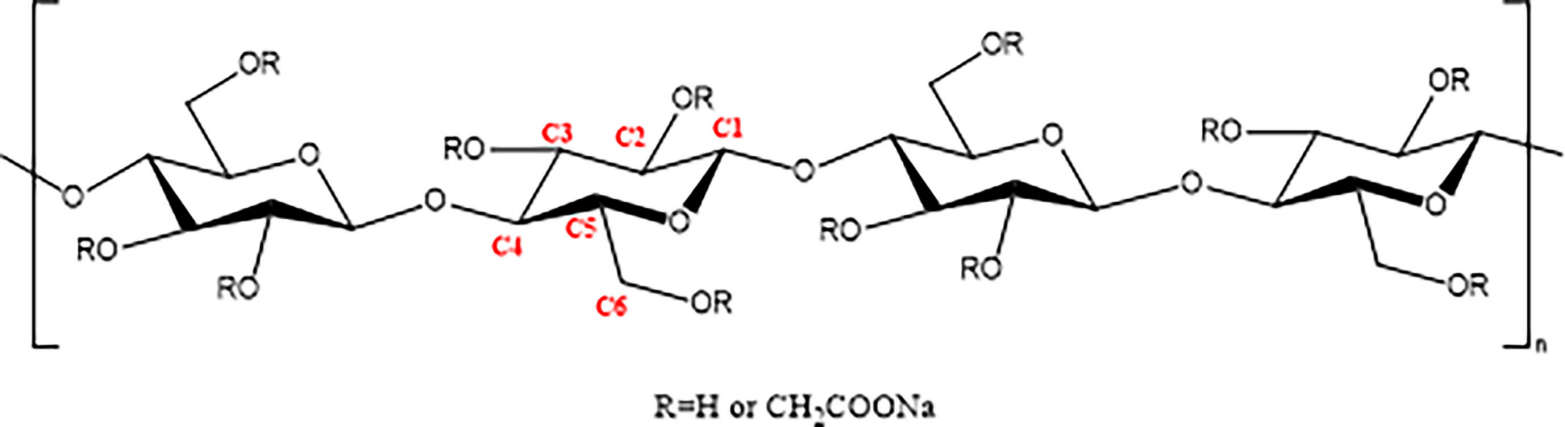
Chemical structure of CMC and carbon ordering in the ring.

Sodium carboxymethyl cellulose (CMC) is a water-soluble cellulose ether material obtained by introducing carboxymethyl groups onto the hydroxyl groups of cellulose chains (figure 1), usually in the form of sodium salt. It has been widely used in various fields such as food, medicine, daily chemicals and the petroleum industry [1,2], typically as a binder, suspending agent or additive to improve performance [3–5]. In recent years, many studies have reported CMC-based loaded materials with coordination effects of carboxyl groups and adsorption capabilities for heavy metal ions or even radioactive nuclides through cross-linking structures formed with other materials [6,7]. They can also be used as drug release control agents [8] or catalyst carriers [9], but most of these loaded materials are CMC composite films [10] or hydrogels [11–13]. Porous foamed materials undoubtedly have great advantages over composite films and hydrogels in terms of adsorption capacity and loading amount owing to their extremely large specific surface area, especially for the loading of heavy metal ions. However, there are currently no reports on the physical and chemical properties of foamed CMC-loaded materials.

Therefore, this study focuses on the characterization and testing of foam produced in a nitric acid system using sodium CMC as the research object. In our laboratory, this foam has been successfully used to load a variety of transition metal ions including some rare earth ions (such as terbium, samarium and praseodymium). Owing to the preparation process of the foam being conducted under strong acidic conditions, it can effectively prevent the hydrolysis of the loaded ions. Infrared spectroscopy, nuclear magnetic resonance (NMR) hydrogen spectroscopy and carbon spectroscopy were used to investigate the interaction between CMC and nitric acid. This provides meaningful scientific references for the study of sodium CMC foam in a nitric acid system.

## 2. Material and methods

### 2.1. General aspects

The sodium CMC, viscosity of 800−1200 mPa s, USP grade and the nitrate (Pr, Sm, Tb, Zr, Ni, analytically pure) were purchased from Aladdin Biochemical Technology Co., Ltd. (Shanghai, China). The nitric acid (65−68%, analytically pure) was purchased from Sinopharm Group Chemical Reagent Co., Ltd. (Shanghai, China), and diluted with deionized water (resistivity > 18.2 MΩ cm) to 2, 3 and 4 mol l^−1^, respectively. Fourier transform infrared spectrometer, Nicolet iS50, Thermo Fisher Scientific Inc. 500 M superconducting NMR spectrometer, Avance III 500, Brulcer CO.

### 2.2. Synthesis of carboxymethyl cellulose foams

**Figure 2.**
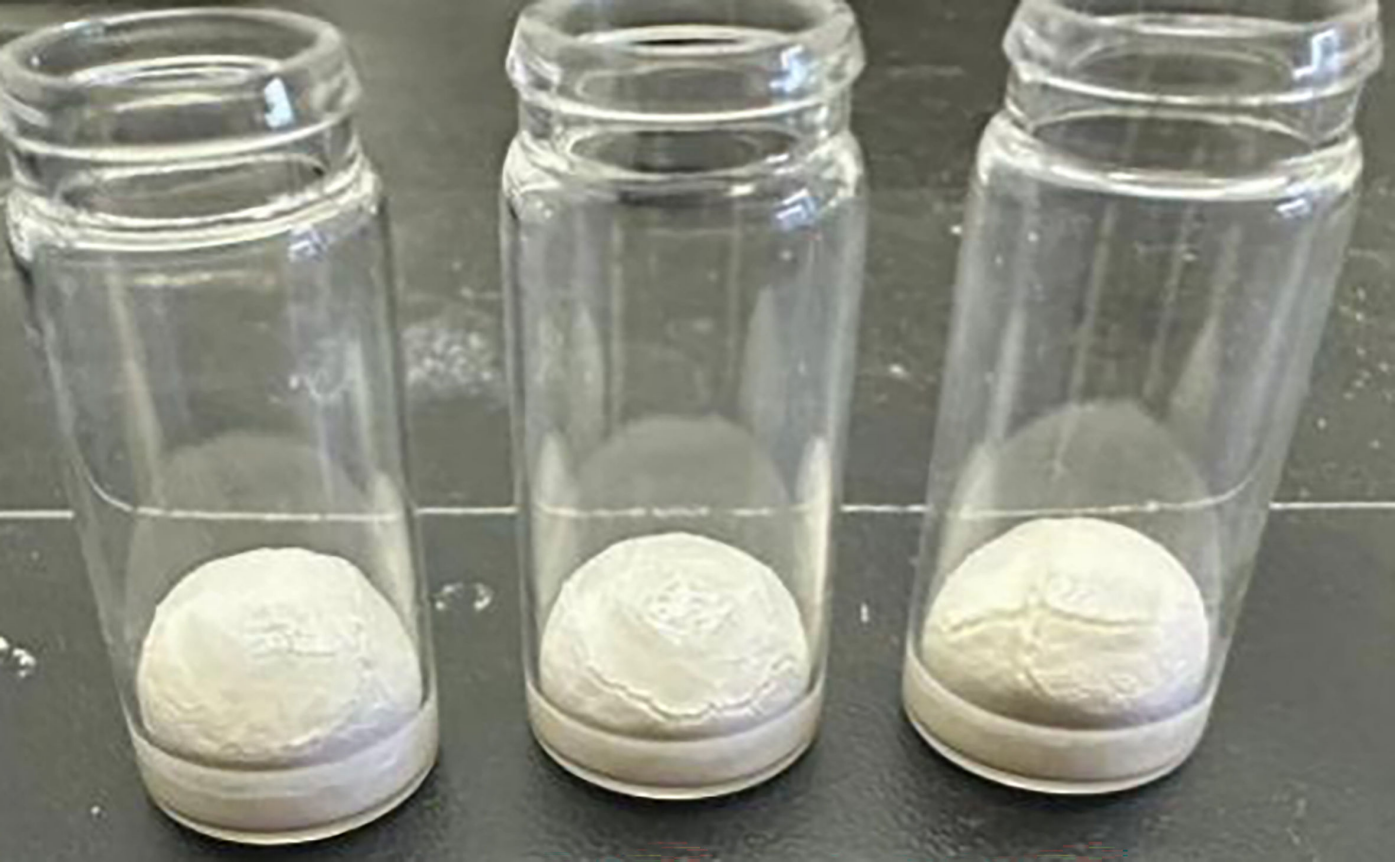
CMC foams made by viscosity ranging from 800 to 1200 mPa s raw material with 3 M HNO_3_.

In total, 25 ml of nitric acid with a concentration of 3 mol l^−1^ was heated to 60°C. Under mechanical stirring at 500 r.p.m., 1.2 g of CMC powder with a viscosity ranging from 800 to 1200 mPa s was slowly added. The stirring continued for 2−3 h to ensure the complete dissolution of the CMC powder. Heating and stirring were stopped when the solution appeared as a clear pale yellow, followed by cooling to room temperature. Aliquots of 2 ml of the prepared CMC nitric acid solution were dispensed into individual 10 ml vials and slowly dried at 55°C for 48 h, resulting in dried CMC foam. Using the same method described above, CMC powder with viscosity ranging from 800 to 1200 mPa s was used to prepare solutions with different concentrations of nitric acid: A_2_(2 mol l^−1^), A_3_(3 mol l^−1^) and A_4_(4 mol l^−1^). This resulted in three different compositions of CMC foam samples (figure 2) labelled according to table 1, where A1 represents the control group using raw material of CMC without nitric acid.

**Table 1.** Compositions of CMC samples.

c(HNO_3_)	0	2 mol l^−1^	3 mol l^−1^	4 mol l^−1^
label	A_1_	A_2_	A_3_	A_4_

### 2.3. Metal ions loaded in carboxymethyl cellulose foams

About 2 ml of CMC nitric acid solution, prepared according to the above method, was slowly added to the nitrate containing approximately 30 mg of different metal ions (terbium, samarium, praseodymium, nickel and zirconium), and allowed to stand for 2 h to ensure complete dissolution of the metal ions in the solution. Subsequently, evaporation was carried out at 55°C for 48 h to obtain different CMC foams loaded with different metal ions.

### 2.4. Sample analysis methods

#### 2.4.1. Fourier transform infrared spectroscopy

The above samples A_1_–A_4_ were prepared into test films using the KBr pellet method. The entire sample preparation process was conducted under an infrared heating lamp to minimize the influence of moisture in the air on sample preparation and measurement. Fourier transform infrared spectroscopy (FTIR) analysis was then performed by scanning 32 times at a resolution of 2 cm^−1^, resulting in transmission spectra. Each group of samples was measured 3−4 times, and only spectra with a maximum transmittance between 40 and 80% were retained for further analysis.

#### 2.4.2. ^1^H-nuclear magnetic resonance and ^13^C-nuclear magnetic resonance

Typically, for cellulose derivatives, the tested samples are completely hydrolyzed into glucose monomers using equal volumes of D_2_SO_4_ and D_2_O before conducting NMR spectroscopy analysis to quantitatively determine parameters such as degree of substitution [14]. However, this method is clearly not feasible for our work owing to the fact that if we use this method, the hydrolysis process of nitric acid with CMC will be covered by D_2_SO_4_ during the preparation of NMR samples, making it impossible to determine the source of active terminal groups. Therefore, in this study, we chose to directly dissolve CMC in D_2_O and then test it: 100 mg A_1_–A_4_ samples were taken separately and added to 5 ml of D_2_O. Ultrasonication was used to completely dissolve them. Then, 0.5 ml from each solution was transferred into NMR tubes for ^1^H-NMR and ^13^C-NMR testing.

## 3. Results and discussion

### 3.1. Successful loading of metal ions

**Figure 3.**
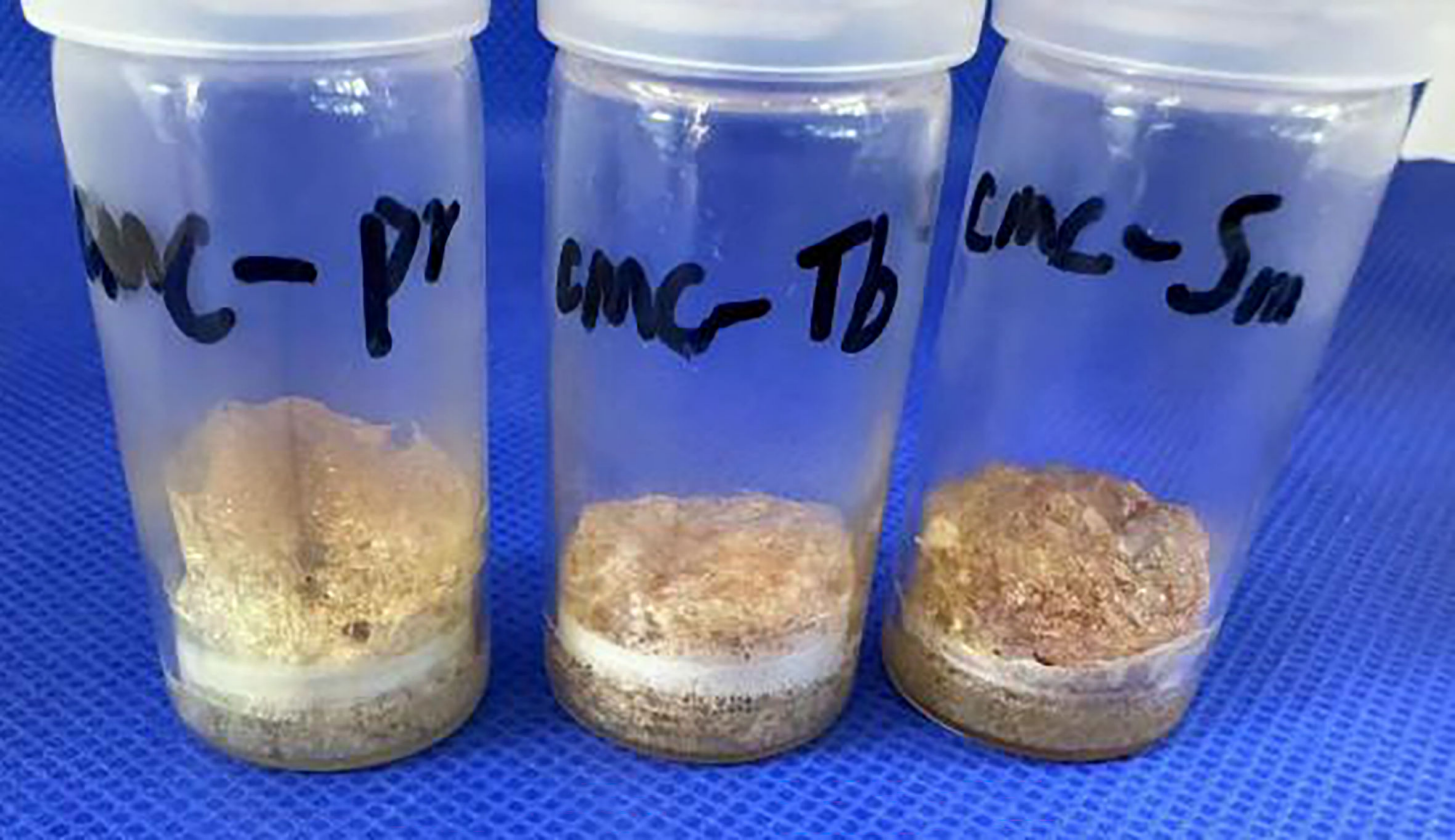
CMC foams loaded with Pr^3+^, Tb^3+^ and Sm^3+^.

**Figure 4.**
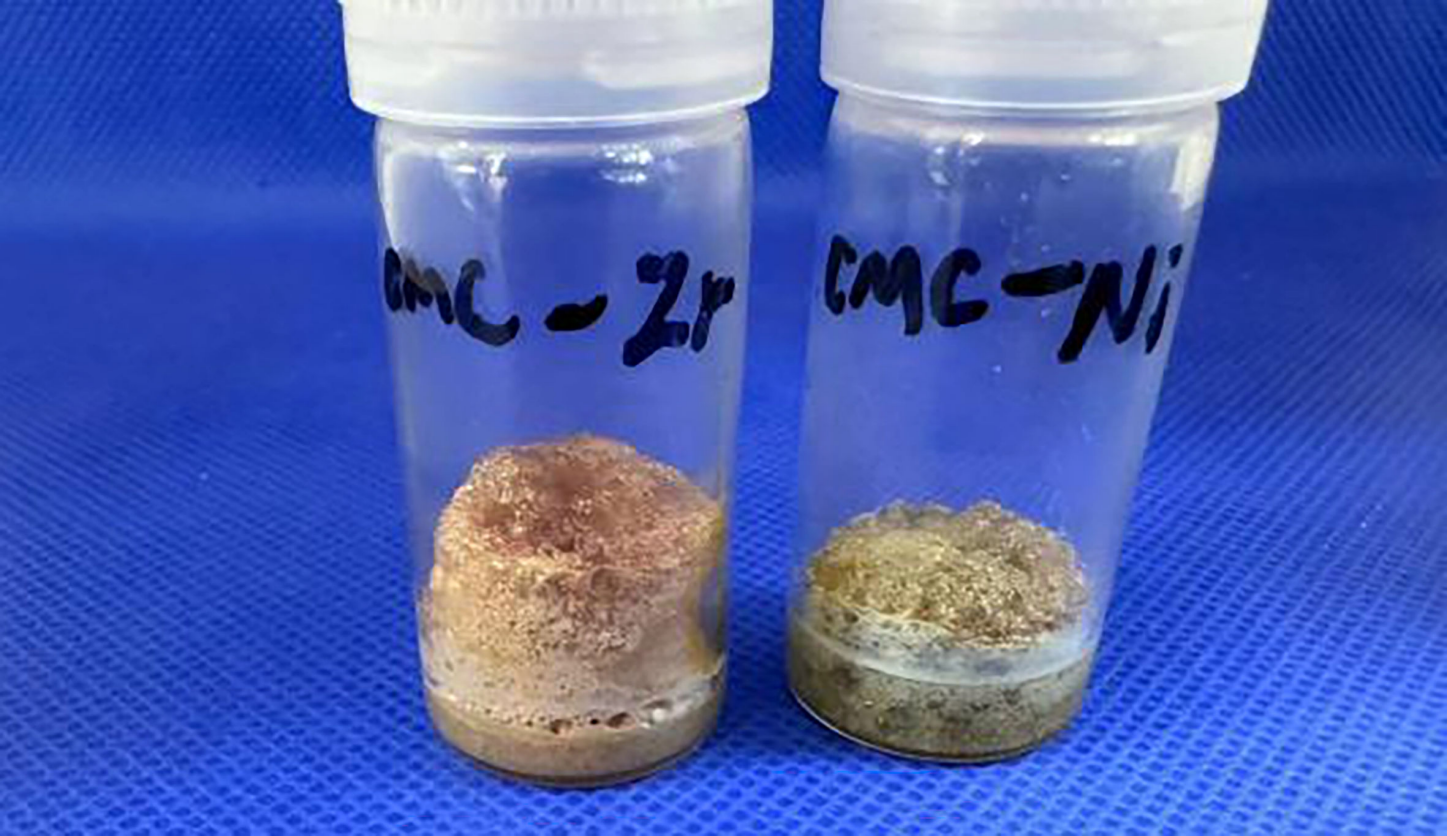
CMC foams loaded with Zr^4+^ and Ni^2+^.

Because CMC materials contain carboxyl groups that give them the properties of polyelectrolytes, these materials are easily flocculated in solution by metal ions, affecting their performance. However, the preparation process is carried out in a strongly acidic environment, which can prevent the precipitation of CMC to some extent [15], allowing it to remain in a viscous gel state even when various metal ions are added, ensuring that the system remains in a viscous gel state throughout the evaporation process. As a result, a good foam-like structure can be formed in the end (figures 3 and 4). There are two main reasons for choosing these metal ions. First, the selected metal ions are all coordinated, and because CMC contains a large number of carbonyl isocoordinated groups, better loading can be achieved. The second reason is to demonstrate the universality of CMC foam loading materials for metal ion loading.

### 3.2. Description of foam formation

**Figure 5.**
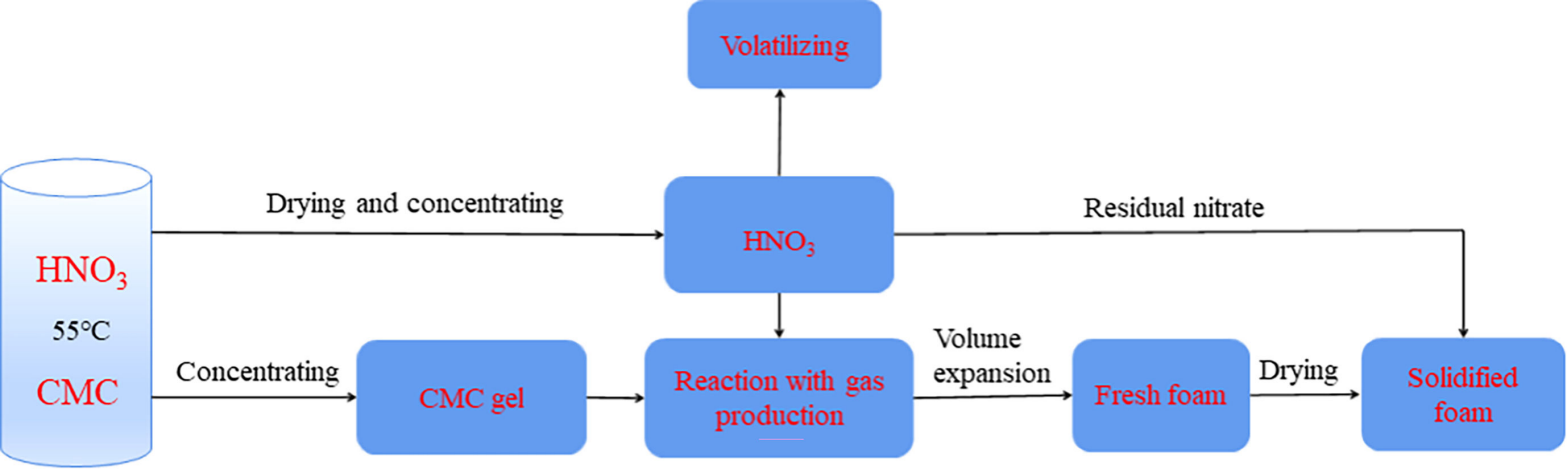
Process of foam formation.

**Figure 6.**
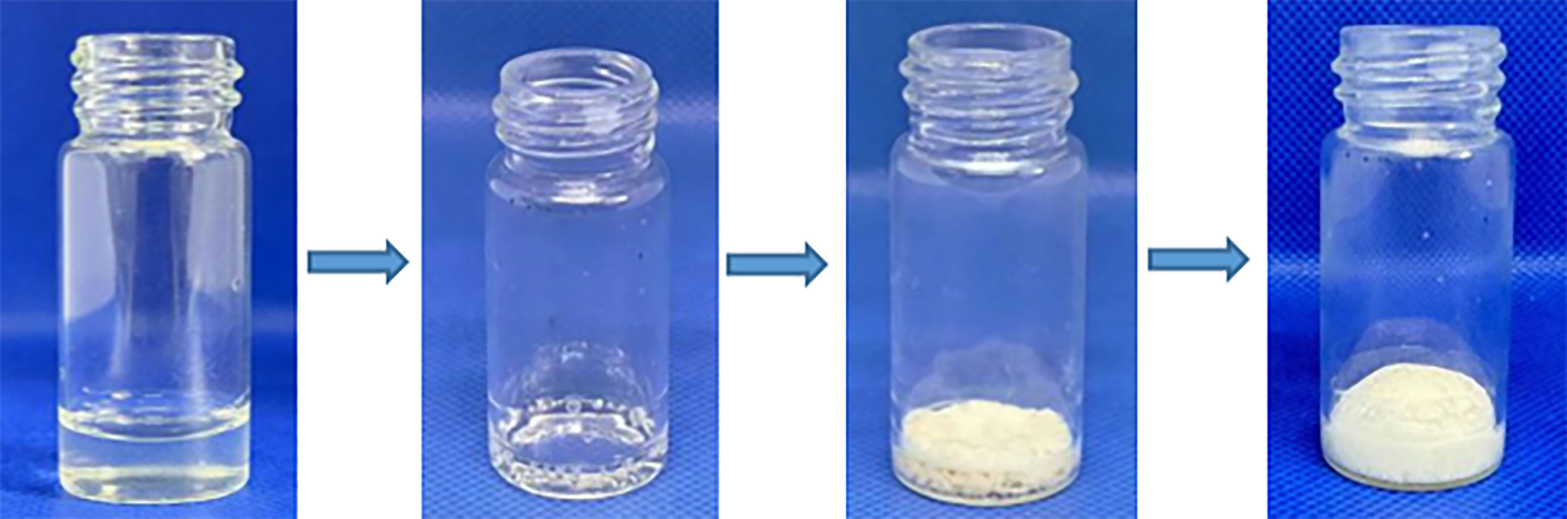
The appearance changes of the foam system at different stages (solution–gel–fresh foam–solidified foam).

According to the experimental phenomenon, the process of foam formation can be divided into three steps, as shown in figures 5 and 6. First, as water evaporates continuously from the vial, the concentration of CMC in the solution increases, forming a viscous gel-like substance. During this process, some nitric acid is also lost owing to volatilization. Second, after evaporation ends, tiny bubbles are generated through the reaction of CMC with nitric acid. Owing to the system’s viscosity, gas production causes continuous expansion of its volume and eventually forms uniform white porous foam. At this stage, the foam is relatively soft, indicating that there is still a significant amount of moisture present in the system. Finally, when the foam volume no longer expands, it indicates that foaming is complete. The remaining moisture inside the system continues to evaporate and harden, resulting in solidified foam.

### 3.3. Fourier transform infrared spectroscopy analysis

**Figure 7.**
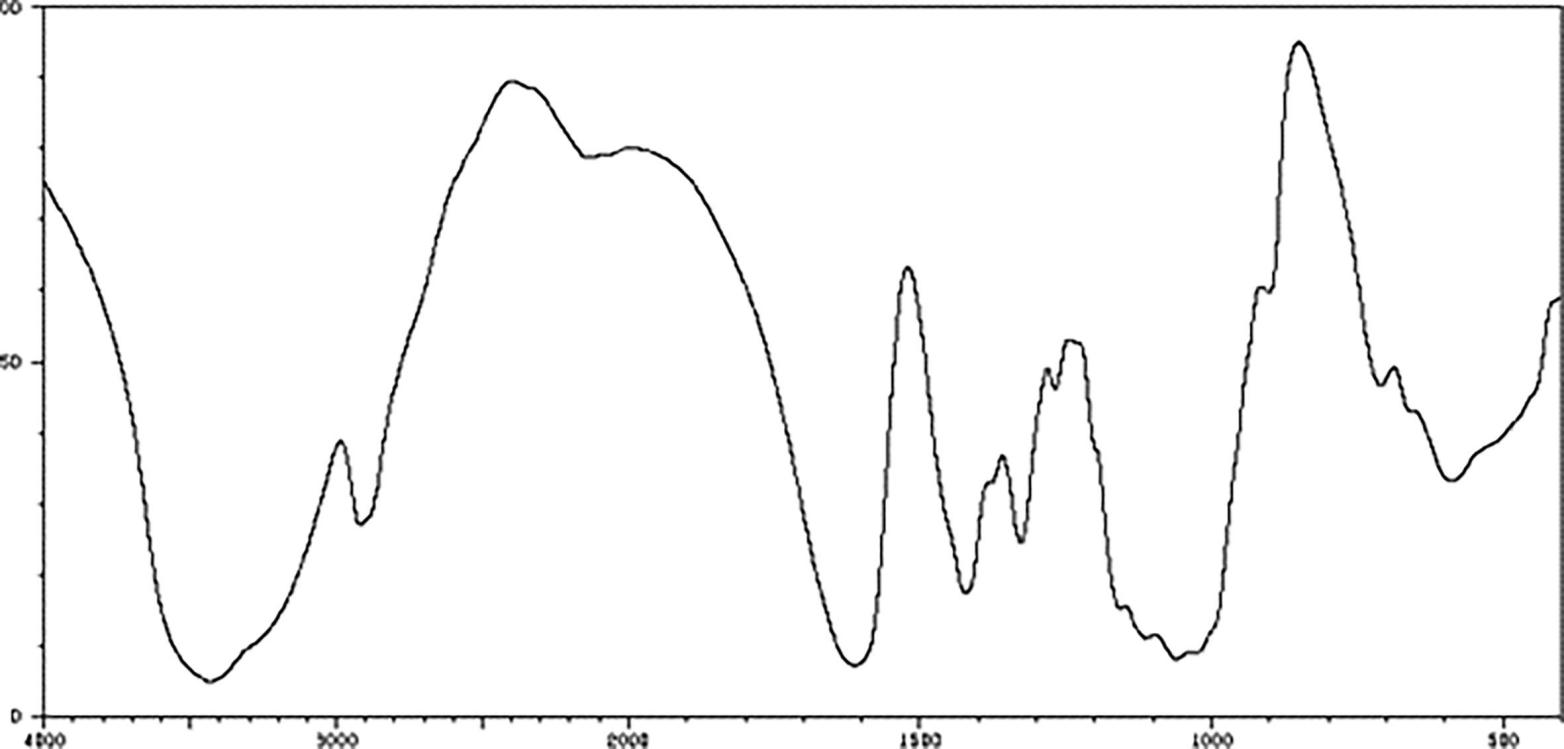
Standard infrared spectrum of CMC (from AIST).

The standard infrared spectrum of sodium CMC is provided in the database of the National Institute of Advanced Industrial Science and Technology (AIST), as shown in figure 7. However, CMC samples produced by different manufacturers and using different production processes may have slight structural variations. Therefore, it is necessary to compare the infrared spectra of different CMC samples with the standard spectrum for analysis. The infrared spectra data for two tested samples are presented in tables 2 and 3.

**Table 2.** FTIR data of CMC raw material.

wavenumber (cm^−1^)	transmittance (%)	attribution
3420	42	−OH and H··O
2928	53	CH_2_
1635	54	COO^−^
1420	54	COO^−^
1010–1195	43	−CH_2_–O–CH_2_−

**Table 3.** FTIR data of CMC foam.

wavenumber (cm^−1^)	transmittance (%)	attribution
3421	54	−OH and H··O
2917	64	CH_2_
1789	62	COOH
1742	57	COOH
1632	64	COO^−^
1384	46	NO_3_^−^
1034–1175	61	−CH_2_–O–CH_2_−

Referring to the research conducted by Riaz *et al*. [16] on the infrared spectrum of CMC, several absorption peaks can be observed in spectrum A_1_: owing to the presence of intermolecular and intramolecular hydrogen bonds and −OH groups, a visible band is seen at 3420 cm^−1^; at 2928 cm^−1^, an absorption peak corresponds to asymmetric stretching vibrations of CH_2_, while peaks at 1420 and 1635 cm^−1^ appear owing to the carboxylate groups stretching vibrations (symmetric and asymmetric); additionally, the region between 1010 and 1195 cm^−1^ represents vibrational modes associated with the cellulose framework (−CH_2_–O–CH_2_−) [17].

**Figure 8.**
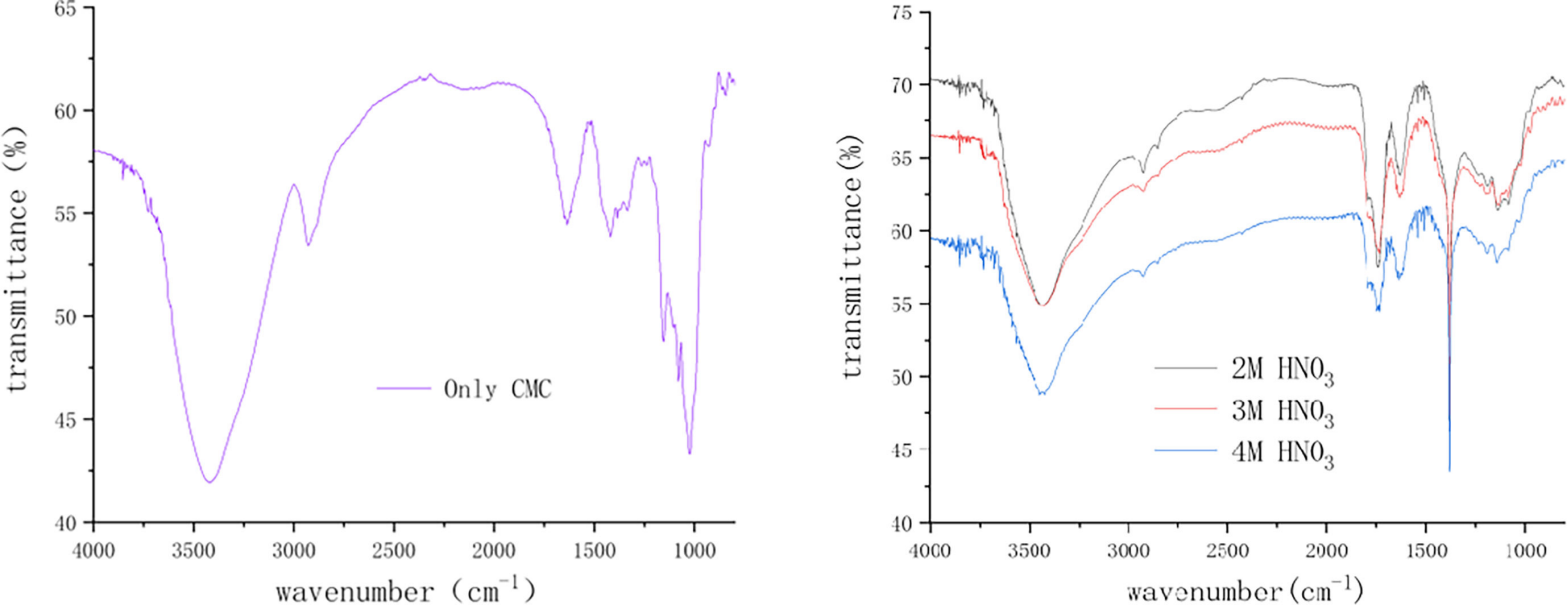
FTIR of CMC raw material (left) and foams with different acidity (right).

**Figure 9.**
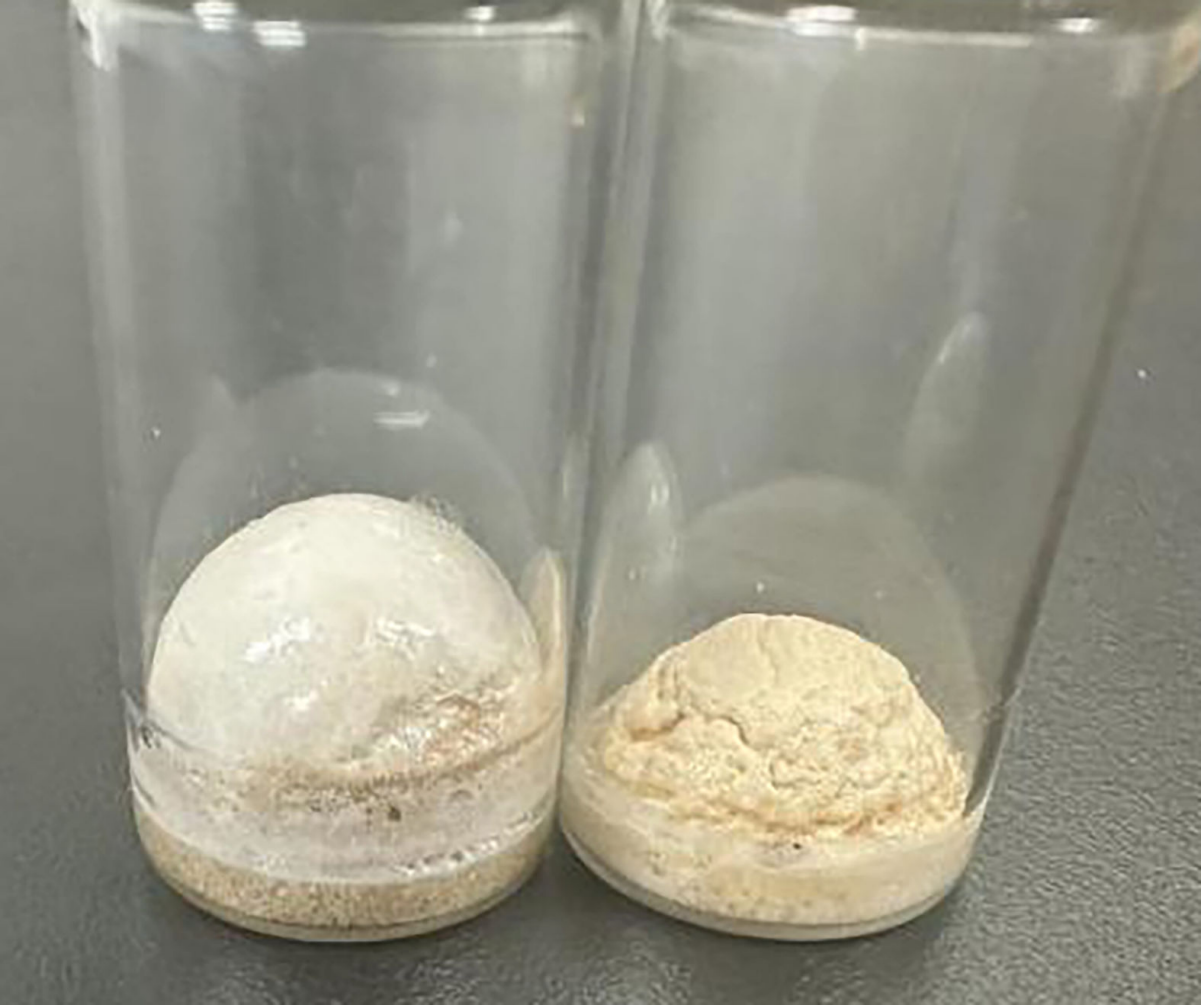
The newly formed foam (left) and after 300 days of room temperature storage (right).

The infrared spectra of A_2_–A_4_ were compared with that of A_1_ (figure 8), revealing two absorption peaks at 1789 and 1742 cm^−1^, associated with carbonyl stretching vibrations. This indicates the acidification of COO^–^ groups in CMC by nitric acid. Additionally, a strong peak at 1384 cm^−1^ corresponds to the characteristic absorption peak of nitrate ions, suggesting a significant amount of residual nitrates in the foam after moulding and drying. This can affect the long-term stability of the foam. The changes in appearance observed after storing the foam for a period of time (figure 9) further support this conclusion. The freshly prepared foam exhibited a full shape but underwent volumetric reduction upon exposure to room temperature for 300 days, indicating the long-term load capacity of the foam. In addition, the absorption strength of the cellulose framework in foam is significantly weakened, which is related to the destruction of the cellulose framework and is associated with its hydrolytic degradation by nitric acid.

### 3.4. ^1^H-nuclear magnetic resonance and ^13^C-nuclear magnetic resonance analysis

**Figure 10.**
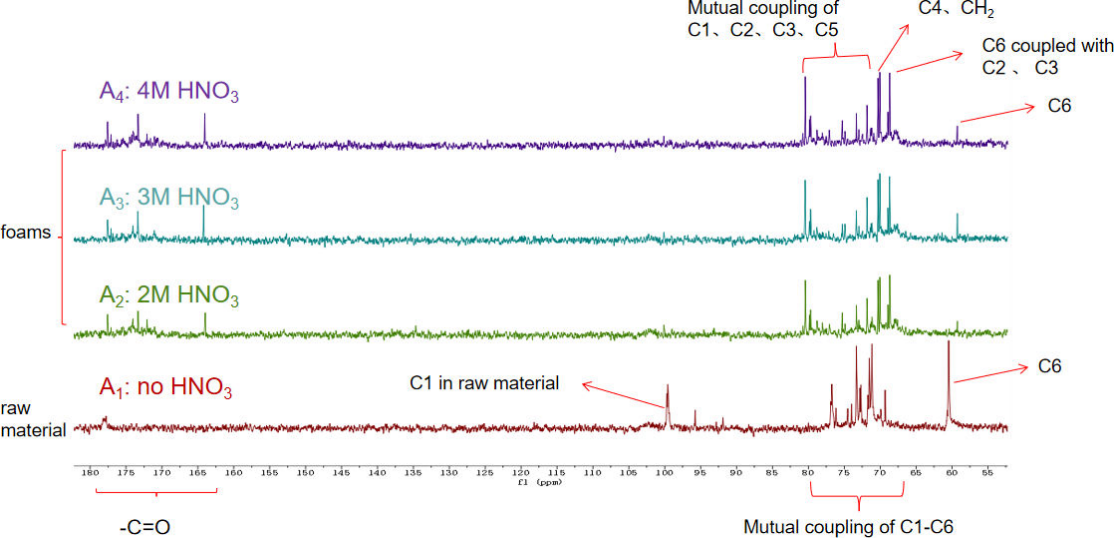
The ^13^C-NMR spectra of foams and raw material (from bottom to top, labelled as A_1_–A_4_).

According to the analysis results of CMC NMR hydrogen and carbon spectra by Nehls *et al*. [17] and Kono *et al*. [18], a brief assignment can be made for the peaks observed in the ^13^C-NMR spectrum (figure 10) of CMC foam and raw materials. In spectra A_2_–A_4_, the peaks at 164–180 ppm are characteristic peaks of carbonyl groups substituted by carboxyl methyl groups at positions C6 (no. 6 carbon in glucose unit, as shown in figure 1), C2 and C3 in the foam structure. The multiple sharp peaks within the range of 71–81 ppm result from coupling between various chemical environments of carbon atoms C2, C3 and C5 on cyclic glucose units. The two sharp peaks at 70.2 and 70.0 ppm correspond to characteristic peaks of C4 and carboxyl methyl group CH_2_, respectively. The peak at 68.6 ppm is a coupling peak between carbon atom C6 with C2 and C3, while the peak at 59.2 ppm represents a characteristic peak of carbon atom C6. In spectrum A_1_, the characteristic peak for carbon atom C1 is observed within the range of 95.5–100.2 ppm in cyclic glucose unit structures.

**Figure 11.**
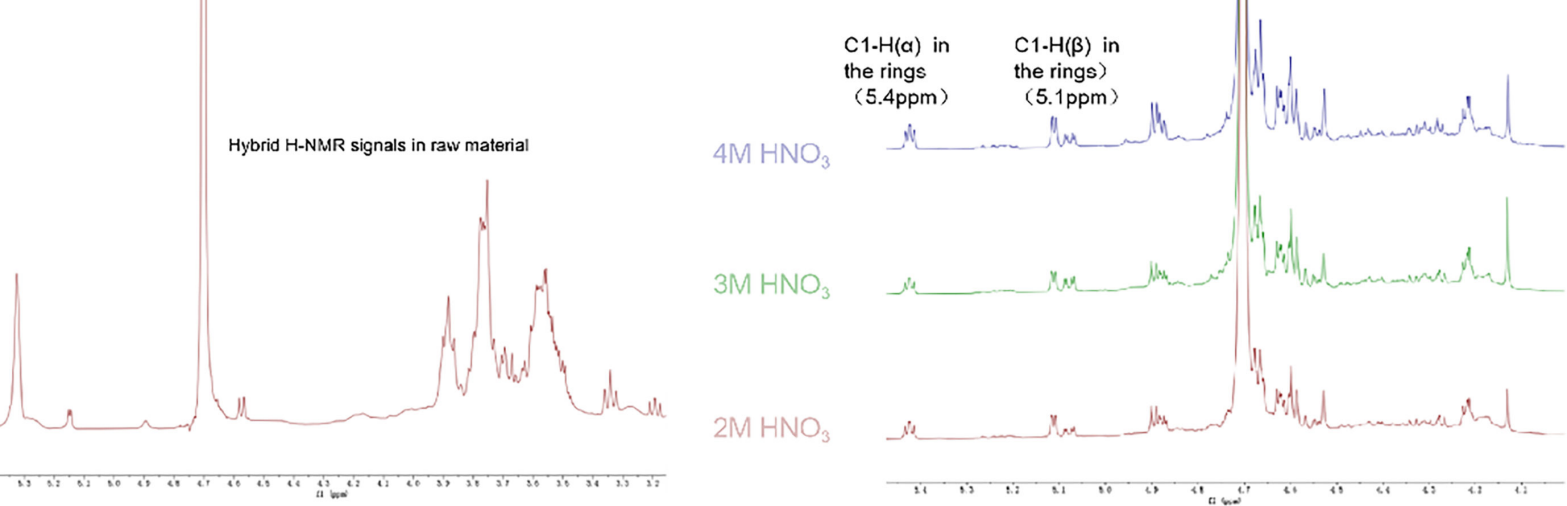
The ^1^H-NMR spectra of raw material (left) and foams (right, from bottom to top, labelled as 2M/3M/4M HNO3).

By comparing ^13^C-NMR peaks in spectra A_2_–A_4_, it can be concluded that there are no significant differences between foam samples prepared under nitric acid concentrations ranging from 2 to 4 mol l^−1^. Furthermore, each carbon’s NMR signal can be clearly identified, indicating that within this concentration range, nitric acid interacts with CMC in a similar manner. Compared to A_1_, the characteristic peak intensity of C1 in the range of 95.5−100.2 ppm for A_2_–A_4_ dramatically decreases and nearly disappears at 4 mol l^−1^, which may be owing to the destruction of C1. However, the ^1^H-NMR spectrum of A_2_–A_4_ (figure 11) still shows two sets of proton resonances for the reducible C1 terminal groups (aldehyde groups) of glucose units at 5.0−5.5 ppm, corresponding to *α* (5.4 ppm) and *β* (5.1 ppm) configurations. This indicates that C1 has not been completely destroyed but changes its state of existence owing to changes in the chemical environment, which also explains the drastic decrease in its signal in the carbon spectrum. The presence of reducible terminal groups suggests that nitric acid hydrolyzes part of the CMC, generating activated reducible C1 terminal groups, which also explains the weakened infrared absorption of the cellulose framework observed in the infrared spectra.

**Figure 12.**
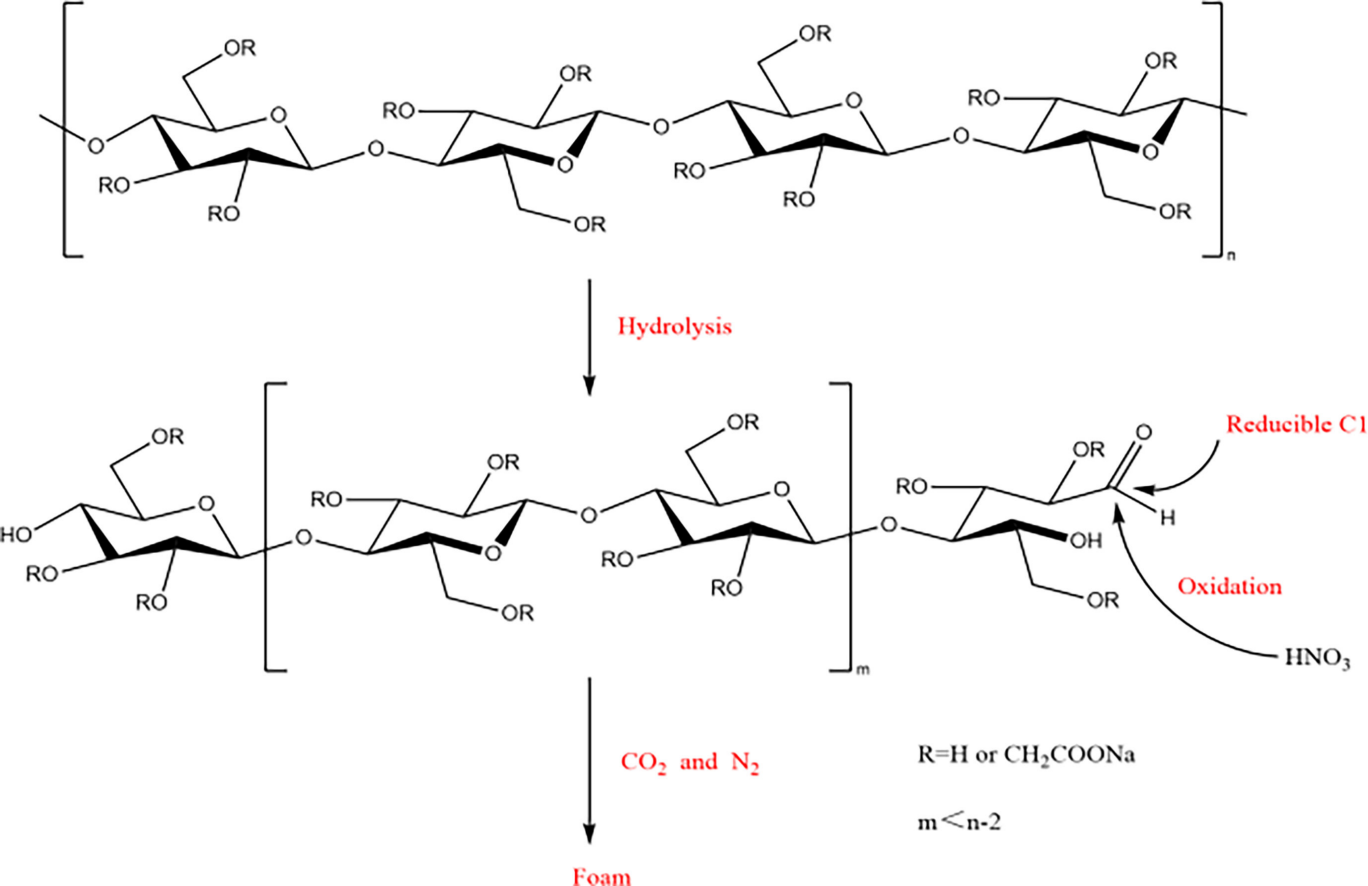
Microscopic mechanism of foam formation.

Owing to the preparation process of foam requiring an open and ventilated environment, it is difficult to quantitatively and qualitatively analyse the types of gases produced. However, it is well known that glucose can undergo oxidation–reduction reactions with concentrated nitric acid, producing CO_2_ and N_2_ gases [19]. In this process, both the hydroxyl and aldehyde groups of glucose are oxidized. It can be inferred that CMC produces similar reducible terminal groups under acidic conditions as nitric acid. However, owing to most hydroxyl groups in CMC being substituted by carboxymethyl groups, they cannot be oxidized by nitric acid. According to the ^13^C-NMR results of CMC foam, carbon signals from C2 to C6 on the glucose unit chain are distinct. Therefore, oxidation mostly occurs at the aldehyde groups (C1) generated through hydrolysis, as shown in figure 12. This ensures relative integrity of the cellulose framework and serves as a prerequisite for foam formation.

## 4. Conclusions

This study presents the preparation process of the foamed CMC-loaded material and elucidates the macro- and microscopic mechanisms of CMC foam formation under nitric acid conditions. This foam is capable of loading a variety of metal ions for over 300 days while maintaining long-term stability. During foam formation, macroscopically, as the solution concentration increases, the system becomes a viscous gel-like substance, followed by the generation of gas from internal oxidation–reduction reactions, causing the system to expand and transform into a foam. Microscopically, the experimental results indicate that within the experimental acidity range, the infrared absorption of the cellulose framework is significantly weakened and the presence of reducible terminal groups indicates that CMC is hydrolyzed into units with a relatively low degree of polymerization. In addition, the carbon spectrum signals from C2 to C6 on glucose monomers do not show obvious changes, suggesting that oxidation mainly occurs at C1, which reacts with increasing concentrations of nitric acid to form foam materials. However, the infrared spectra indicate a significant amount of nitrate in the foam system, and the foams undergo volumetric reduction over an extended period of storage time. Therefore, it is necessary to reasonably control the amount of nitric acid during foam preparation to prevent excessive damage to the structure of CMC foam. This provides a theoretical foundation for further optimizing the preparation process for CMC foam.

## Data Availability

All data generated and analysed during the current study are included in the manuscript, and the original data has been uploaded on Dryad datasets [20].
